# Large Controlled Observational Study on Remote Monitoring of Pacemakers and Implantable Cardiac Defibrillators: A Clinical, Economic, and Organizational Evaluation

**DOI:** 10.2196/ijmr.4270

**Published:** 2016-01-13

**Authors:** Claudio Dario, Pietro Delise, Lorenzo Gubian, Claudio Saccavini, Glauco Brandolino, Silvia Mancin

**Affiliations:** ^1^ Arsenàl.IT, Veneto's Research Centre for eHealth Innovation Treviso Italy; ^2^ Department of Cardiology, General Hospital of Conegliano Conegliano Italy; ^3^ Veneto Region Health Information System, Italy Venice Italy

**Keywords:** healthcare economics and organization, telehealth and telemonitoring, implantable cardiac defibrillator, cardiac pacemaker

## Abstract

**Background:**

Patients with implantable devices such as pacemakers (PMs) and implantable cardiac defibrillators (ICDs) should be followed up every 3–12 months, which traditionally required in-clinic visits. Innovative devices allow data transmission and technical or medical alerts to be sent from the patient's home to the physician (remote monitoring). A number of studies have shown its effectiveness in timely detection and management of both clinical and technical events, and endorsed its adoption. Unfortunately, in daily practice, remote monitoring has been implemented in uncoordinated and rather fragmented ways, calling for a more strategic approach.

**Objective:**

The objective of the study was to analyze the impact of remote monitoring for PM and ICD in a “real world” context compared with in-clinic follow-up. The evaluation focuses on how this service is carried out by Local Health Authorities, the impact on the cardiology unit and the health system, and organizational features promoting or hindering its effectiveness and efficiency.

**Methods:**

A multi-center, multi-vendor, controlled, observational, prospective study was conducted to analyze the impact of remote monitoring implementation. A total of 2101 patients were enrolled in the study: 1871 patients were followed through remote monitoring of PM/ICD (I-group) and 230 through in-clinic visits (U-group). The follow-up period was 12 months.

**Results:**

In-clinic device follow-ups and cardiac visits were significantly lower in the I-group compared with the U-group, respectively: PM, I-group = 0.43, U-group = 1.07, *P*<.001; ICD, I-group = 0.98, U-group = 2.14, *P*<.001. PM, I-group = 0.37, U-group = 0.85, *P*<.001; ICD, I-group = 1.58, U-group = 1.69, *P*=.01. Hospitalizations for any cause were significantly lower in the I-group for PM patients only (I-group = 0.37, U-group = 0.50, *P*=.005). There were no significant differences regarding use of the emergency department for both PM and ICD patients. In the I-group, 0.30 (PM) and 0.37 (ICD) real clinical events per patient per year were detected within a mean (SD) time of 1.18 (2.08) days. Mean time spent by physicians to treat a patient was lower in the I-group compared to the U-group (-4.1 minutes PM; -13.7 minutes ICD). Organizational analysis showed that remote monitoring implementation was rather haphazard and fragmented. From a health care system perspective, the economic analysis showed statistically significant gains (*P*<.001) for the I-group using PM.

**Conclusions:**

This study contributes to build solid evidence regarding the usefulness of RM in detecting and managing clinical and technical events with limited use of manpower and other health care resources. To fully gain the benefits of RM of PM/ICD, it is vital that organizational processes be streamlined and standardized within an overarching strategy.

## Introduction

Patients with implantable devices such as pacemakers (PM) and implantable cardiac defibrillators (ICD) should be followed up every 3–12 months, which traditionally required in-clinic visits. Innovative devices allow data transmission and technical or medical alerts to be sent from the patient's home to the physician. This is known as remote monitoring (RM). Recent studies have shown the clinical benefits of remote monitoring of cardiac implantable electronic devices (CIEDs) [1-9]. As a consequence, a number of scientific societies [10,11] have integrated the RM of CIED within their guidelines. However, the adoption of RM of CIED by several European health care services is still somewhat patchy [12,13]. Hurdles must be overcome before large scale RM can become routine [13,14]. Above all, scaling up remote patient monitoring requires effective strategies to address clinical, technological, organizational, economic, and ethical dimensions. Within the framework of the European RENEWING HEALTH project [15], an observational, prospective study, unfunded by device vendors, was implemented. The study adopted a rigorous assessment approach (model for assessment of telemedicine, MAST) [16], as an overall framework.

## Methods

### Study Objectives

The study analyzed the effectiveness and efficiency of RM for pacemakers (PM) and implantable cardiac defibrillators (ICD) in a “real world” context compared with in-clinic follow-up. The following outcomes were considered: specialist visits (in-clinic PM/ICD follow-ups, cardiology visits), hospital admissions for any cause, accesses to the emergency department, timeliness of detection of acute episodes recorded by the device, workload, and direct costs.

### Study Protocol

This study is a multi-center, multi-vendor, controlled, observational, prospective study. Patients were enrolled by six cardiology departments located within six different local health authorities (LHAs). Currently, each cardiology unit follows more than 1900 patients with an implanted device. We assumed both cardiology units and communities, which reside within different LHAs, but belong to the same northern Italian region (Veneto), to be similar.

There were five LHAs that assigned patients to the I-group and followed them up with a RM system. These patients were enrolled during in-clinic follow-up, either after device implantation or directly invited to participate in the study. In the I-group, patients with a PM were not monitored through in-clinic follow-ups, unless necessary from a clinical or technological point of view. Patients with an ICD were offered at least one in-clinic follow-up. A sixth LHA registered consecutive patients as a control group (U-group) during routine follow-ups. This LHA had no experience with CIED RM. U-group patients were followed up through regularly scheduled in-clinic visits. Follow-ups were performed every 12 months for PM and every 6 months for ICD, and any variation in visits’ frequency was related to CIED functioning.

Inclusion criteria were: patients with implanted PM and ICD devices; patients who had given written consent to participate in the study; age > 18; not pregnant; and absence of comorbidities with a life expectancy < 12 months. Both patient groups were followed up for 12 months. Local Ethics Committees have approved the study protocol in accordance with the Declaration of Helsinki.

### Remote Monitoring Service

**Figure 1:**
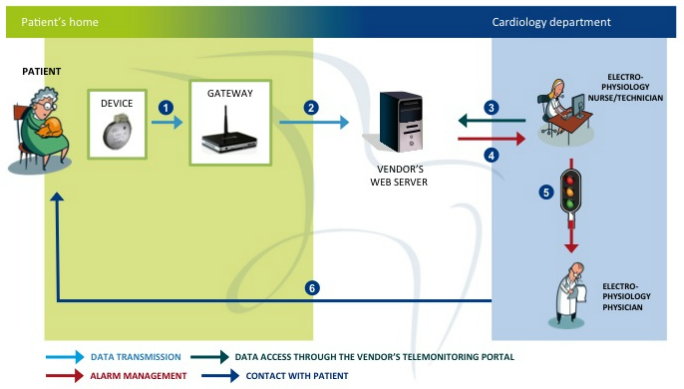
Workflow of remote monitoring service [15].

RM systems included both wireless RM (WRM) and manual RM devices. WRM devices enable the automatic transmissions of daily or weekly alerts, whereas manual RM devices require that patients manually interrogate the PM/ICD with the handle of the gateway. Centers involved in the study used a similar organizational model to provide telemedicine services. Figure 1 shows the workflow for managing RM of PM/ICD patients.

The process consists of the following six steps:

1. PMs and ICDs periodically relay remote programmed transmissions (RPTs), and daily or weekly transmit serious recorded events to a home gateway;
2. The gateway automatically sends data to the vendor’s Web server;
3. The nurse checks RPTs’ data daily during regular working time, accessing them through the different vendors’ Web-portals;
4. In case of an alert, the nurse receives a notification via email, fax, or short message service, and, still during regular working time, reviews data;
5. In case of a serious event, the nurse submits data to the physician. The physician evaluates data, and decides if the patient needs a specialist visit, in-clinic device follow-up, therapy modification, or other actions; and
6. When appropriate, the nurse contacts the patient to offer recommendations and care instructions.

All involved nurses and physicians had specific competence in cardiac electrophysiology and electro-stimulation. They were also exposed to a specific training offered by vendors’ specialists regarding the use of RM technology and portals. The training consisted of a face-to-face session lasting one hour. RM systems [17] were supplied by one of the five following Companies: (1) HM of Biotronik Gmbh, Berlin, German; (2) CareLink Network by Medtronic Inc, Minneapolis, MN, USA; (3) Latitude Patient Management System by Boston Scientific, St Paul, MN, USA; (4) Merlin.Net system by St Jude Medical, Sylmar, CA, USA; and (5) SmartView system by Sorin Group, Italy.

### Data Collection and Evaluation

At enrollment, patient sociodemographic and clinical data at the time of the implant were collected through a case report form (CRF). Data concerning health care services’ use (exams, outpatient visits, visits to the emergency department, hospitalizations, medications supplied by hospitals and pharmacies) were extracted from the Veneto region data-warehouse. For WRM systems, data on type of event, reaction time, and clinical decisions were collected. Events generated by devices were separated into real and false positive events, for example, not useful for patients’ management. Clinical events included: ventricular tachycardia, supraventricular tachycardia, thoracic impedance out of range suggesting pulmonary edema, effects of therapy delivered by the device, and others. Technical events consisted of: low battery, malfunctioning of leads (sensing, threshold, impedance and disabling of auto-capture), low percentage of left ventricular pacing, high percentage of right ventricular pacing, inappropriate shock, and others. Events related to flawed communication between device and vendors’ servers were also collected.

As an overall framework, this study adopted the MAST [16] model. However, due to limitations of data concerning the patient perspective, our economic analysis was limited to the health care system’s perspective. Theoretically, the value of the resources to be considered is their opportunity cost, but since this is often difficult to estimate, a pragmatic approach recommends the use of market prices. Staff costs were bases on the average “total employee cost” of health professionals involved in the study, including their gross compensation, severance indemnity and Social Security allowances, and health insurance. Diagnosis related group (DRG) payment rates were used to quantify the costs of health care services delivery. Such costs are listed in the Veneto Regional Health Service Register of Tariffs, together with costs incurred by the Regional Pharmaceutical Health care system. Remote follow-ups generate extra costs related to the additional services provided by the device manufacturers and to the involvement of health care professionals who monitor patients’ data. Currently, the former does not represent a marginal cost, since in Italy vendors do not require a fee for implementing RM service. This is why in our study, similarly to other recent analyses [18], the cost of the RM service was not considered. Given that remote follow-up is not covered by official reimbursement, its cost was estimated on the basis of time consumed by RM management. The vendors did not charge for the gateway and server acquisition. There were no other hardware investment costs.

Data concerning organizational aspects were collected through the following tools: semistructured interviews of clinicians, CRF of alerts, and regional data-warehouse. Total time spent caring for patients in the I-group was estimated by adding the time needed to deliver in-clinic follow-ups and to manage telemedicine services. For both groups, time spent to provide in-clinic follow-ups was collected, differentiating between nurses and physicians.

### Statistical Analysis

The analysis of the cost items revealed some outliers. Therefore, the interquartile range (IQR) was used to sort the data. Costs below the lower fence (1° quartile - 1.5 IQR) and above the upper fence (3° quartile + 1.5 IQR) were considered as outliers and excluded from the analysis. Cost variables were considered normally distributed [19]. Normality of outcomes data was assessed using the Shapiro-Wilk W test. When variables were not normally distributed, groups were compared using the Mann-Whitney’s U test. Differences between groups are displayed as difference of means, medians with 95% confidence intervals, and IQR. For categorical clinical outcomes, we used chi-square test of goodness of fit or the Fisher test. Differences between groups are calculated as risk ratios with 95% confidence intervals. The analysis was carried out using R software 3.0.1.

Standard data quality control tools, such as data entry controls including tolerance limits, ranges for applicable data fields, and data sequence checks, were used. Clinicians involved in the study had adequate competence and followed the study protocol. Controlled access to survey results and data-warehouse, including password protection and locking formulas, and documented processes ensuring appropriate timing and frequency of data back-up, were used.

## Results

### Patient Population

On the whole, 1871 (979 PM, 892 ICD) patients were enrolled in the I-group and 230 (192 PM, 38 ICD) in the U-group, from October 2011 to November 2012. There were no important differences between the two groups (Table 1). The population resembled the expected characteristics of CIED patients, being comparable to the largest and most recent Italian CIED registry [7]. There were (2.03%) 38/1871 patients allocated to the I-group that were lost to follow-up. In the PM group, loss to follow-up was (2.0%) 20/979, due to: choice of patients or relatives (7); technical difficulties in the use of RM systems (7); moving to another LHA (4); and other (2). In the ICD group, loss to follow-up was (2.0%) 18/892, due to: choice of patients or relatives (5); technical difficulties in the use of RM systems (8); moving to another LHA (2); and other (3). There was no significant difference in mortality between the I-group and the U-group PM: I-group (6.6%) 63/959 deaths, U-group (4.4%) 8/184 deaths, and *P*=.27; and ICD: I-group (5.4%) 47/874 deaths, and U-group (5%) 2/38 deaths, and *P*=.99. Patients who completed the study comprised: 896 PMs (419 with WRM function) and 827 ICDs (811 with WRM function) in the I-group, and 184 PMs and 36 ICDs in the U-group.

### Clinical Evaluation

In the PM group, the mean (IQR) of hospitalizations per patient-year was 0.37 (0-0) in the I-group versus 0.50 (0-1) in the U-group (*P*=.005). The mean (IQR) of in-clinic follow-ups per patient-year was 0.43 (0-1) in the I-group versus 1.07 (1-1) in the U-group (*P*<.001). The mean (IQR) number of cardiology visits per patient-year was 0.37 (0-1) in the I-group versus 0.85 (1-1) in the U-group (*P*<.001). There were no significant differences regarding the number of visits to the emergency department in the I-group, 0.64 (0-1), compared to the U-group, 0.67 (0-1).

In the ICD group, there were significant differences in the in-clinic follow-ups and cardiology visits. The mean (IQR) number of in-clinic follow-ups per patient-year was statistically different (*P*<.001): the I-group 0.98 (0-2) versus the U-group 2.14 (2-2.25). The mean (IQR) number of cardiology visits per patient per year was also statistically significant (*P*=.01): the I-group 1.58 (0-2) versus the U-group 1.69 (1-2.25). However, there were no significant differences regarding the number of hospitalizations per patient per year between the I-group 0.60 (0-1) and the U-group 0.67 (0-1) and in the number of visits to the emergency department 0.80 (0-1) in the I-group versus the U-group 0.64 (0-1).

In the PM group, a total of 125 real clinical events (0.30 events per patient per year) were detected within a mean (SD) time of 1.18 (2.08) days. In the ICD group, a total of 300 real clinical events (0.37 events per patient per year) were found within a median (SD) time of 1.03 (1.68) days. There were 21.9% and 21.7% of patients that presented at least one real clinical event in both the PM (92 of 419) and the ICD (176 out of 811) group. Among patients, without history of atrial fibrillation (AF), enrolled at implant or within the first days after implant, the percentage of first detected episodes of AF was 17% (13 out of 75) in the PM group, and 10.7% (13 out of 122) in the ICD group.

### Economic Evaluation

Tables 2 (PM) and 3 (ICD) show the mean direct costs per patient per year. Economic results are statistically significant only for the PM-group (*P*<.001).

**Table 1:** Baseline data.

Measurements			I-group PM	U-group PM	I-group ICD	U-group ICD
Sample size (n)			979	192	892	38
Age at enrollment (years), mean (SD)			76.93 (10.75)	77.85 (8.49)	67.45 (13.46)	66.66 (11.24)
Age at implant (years), mean (SD)			75.36 (10.78)	76.34 (8.54)	65.83 (13.28)	64.80 (11.6)
Men, n (%)			588 (60.1)	100 (52.1)	708 (79.4)	30 (79)
New implant, n (%)			700 (71.5)	153 (79.7)	573 (64.1)	30 (79)
Replacement, n (%)			279 (28.5)	39 (20.3)	320 (35.9)	8 (21)
**Type of device, n (%)**						
	Single-chamber		248 (25.3)	86 (44.8)	342 (38.3)	15 (40)
	Dual-chamber		679 (69.4)	103 (53.6)	193 (21.6)	7 (18)
	Biventricular		52 (5.3)	3 (1.6)	358 (40.1)	16 (42)
**Implant indication, n (%)**						
	Atrium-ventricular block		493 (50.3)	79 (41.1)	n/a	n/a
	Sick sinus syndrome		136 (13.9)	43 (22.4)	n/a	n/a
	Syncope		122 (12.5)	20 (10.4)	n/a	n/a
	Heart failure		39 (4.0)	2 (1.0)	n/a	n/a
	Bradicardia atrial fibrillation		121 (12.4)	40 (20.8)	n/a	n/a
	Other		13 (1.3)	6 (3.1)	n/a	n/a
	Data missed		55 (5.6)	2 (1.0)	2 (0.2)	n/a
	Primary prevention		n/a	n/a	666 (74.7)	25 (66)
	Secondary prevention		n/a	n/a	224 (25.1)	13 (34)
Ejection fraction, % (SD)			55.4 (11.0)	60 .1(9.7)	35.4 (11.5)	37.1 (11.3)
**New York Heart Association class, n (%)**						
	I		636 (65.0)	126 (65.6)	247 (27.6)	16 (42)
	II		258 (26.4)	55 (28.7)	431 (48.3)	16 (42)
	III		67 (6.8)	10 (5.2)	197 (22.1)	5 (13)
	IV		8 (0.8)	0 (0.0)	14 (1.6)	0 (0)
	Data missed		10 (1.0)	1 (0.5)	3 (0.4)	1 (3)
**Cardiovascular disease, n (%)**						
	AMI		103 (10.5)	25 (13.0)	375 (42.0)	17 (45)
	Hypertension		539 (55.1)	117 (60.9)	378 (42.3)	7 (18)
	Heart failure		91 (9.3)	16 (8.3)	350 (39.2)	14 (37)
	Ventricular arrhythmia		13 (1.3)	1 (0.5)	298 (33.4)	11 (29)
	**Atrial arrhythmia**		340 (34.7)	70 (36.5)	235 (26.3)	10 (26)
		Atrial fibrillation	302 (30.8)	62 (32.3)	222 (24.9)	9 (24)
		Other atrial arrhythmias	38 (3.9)	8 (4.2)	14 (1.5)	1 (3)
	Dilated cardiomyopathy		n/a	n/a	157 (17.6)	3 (8)
			n/a	n/a	29 (3.2)	1 (3)
	Brugada syndrome		n/a	n/a	14 (1.6)	0 (0)
	Arrhythmogenic right ventricular dysplasia		n/a	n/a	20 (2.2)	0 (0)
	Other cardiomyopathies		207 (21.1)	19 (9.9)	162 (18.2)	0 (0)
	None		214 (21.9)	23 (12.0)	7 (0.8)	0 (0)
	Data missing		33 (3.4)	2 (1.0)	11 (1.2)	0 (0)

**Table 2:** Mean direct costs of care per PM patient per year (€, 2011 prices).

Type of cost			Mean direct cost per patient - PM group				Mean difference, €	Confidence interval	*P*
			U-group, €	Confidence interval	I- group, €	Confidence interval			
**Investment in the telemedicine application**									
	**Project start up costs**								
		Nurses' training	—	n/a	1.33	1.18-1.46	1.33	n/a	n/a
		Technicians' training	—	n/a	0.12	0.10-0.13	0.12	n/a	n/a
		Specialists' training	—	n/a	4.90	4.60-5.19	4.90	n/a	n/a
		Total start up costs	—		6.35		6.35		
**Running costs**									
	**Travel**								
		Cost of transportation of the patient and caregiver to the hospital for outpatient visits and procedures (borne by LHA)	1.41	-2.30 to 6.92	8.47	-0.87 to 4.10	7.06	-5.92 to 4.52	.79
	**Staffing**								
		Nurses' time used for patient training	—	n/a	4.51	n/a	4.51	n/a	n/a
		Nurses'/technicians' time used for RM	—	n/a	5.51	5.35-5.65	5.51	n/a	n/a
		Specialist's time used for RM	—	n/a	5.21	4.95-5.46	5.21	n/a	n/a
	**Changes in the use of health care resources**								
		Outpatient visits and procedures	312.80	283.95-351.20	335.09	317.33-352.86	22.29	-20.45 to 55.48	.36
		Emergency room admissions	50.08	36.49-63.66	38.19	32.97-43.39	-11.89	-26.42 to 2.63	.11
		Hospitalizations in acute hospitals	816.47	601.18-1031.75	0	n/a	-816.47	-1031.74 to -601.18	<.001
		Medications (distributed by the hospital)	—	n/a	—	n/a	—	n/a	n/a
		Medications (distributed by pharmacies)	595.07	536.41-653.73	569.09	541.44-596.74	-25.98	-90.75 to 38.78	.43
Total running costs			1775.83		966.07		-809.76		
Total costs			1775.83	1545.41-2007.28	972.42	938.15-1009.12	-803.41	-1036.32 to -569.11	<.001

**Table 3:** Mean direct costs of care per ICD patient per year (€, 2011 prices).

Type of cost			Mean direct cost per patient - ICD group				Mean difference, €	Confidence interval	*P*
			U-group, €	Confidence interval	I- group, €	Confidence interval			
**Investment in the telemedicine application**									
	**Project start up costs**								
		Nurses' training	—	n/a	1.57	1.51-1.64	1.57	n/a	n/a
		Technicians' training	—	n/a	0.16	0.14-0.18	0.16	n/a	n/a
		Specialists' training	—	n/a	5.54	5.32-5.75	5.54	n/a	n/a
		Total investment costs	—		7.27		7.27		
**Running costs**									
	**Travel**								
		Cost of transportation of the patient and caregiver to the hospital for outpatient visits and procedures (borne by LHA)	0	n/a	3.16	n/a	3.16	n/a	n/a
	**Staffing**								
		Nurses' time used for patient training	—	n/a	4.51	n/a	4.51	n/a	n/a
		Nurses'/technicians' time used for RM	—	n/a	8.59	8.34-8.84	8.59	n/a	n/a
		Specialist's time used for RM	—	n/a	7.94	7.62-8.25	7.94	n/a	n/a
	**Changes in the use of health care resources**								
		Outpatient visits and procedures	473.88	391.99-555.76	434.30	411.25-457.33	-39.58	-124.40 to 45.23	.35
		ER admissions	20.09	-0.43 to 40.60	46.96	40.14-53.78	26.87	5.35-48.39	.02
		Hospitalizations in acute hospitals	866.94	155.15-1578.73	572.13	475.80-668.44	-294.81	-1012.46 to 422.83	.41
		Medications (distributed by the hospital)	0.02	0.02-0.073	1.39	0.99-1.79	1.37	0.96-1.77	<.001
		Medications (distributed by pharmacies)	758.26	605.01-911.52	726.86	686.50-755.22	-31.40	-194.16 to 119.35	.63
Total running costs			2119.19		1805.84		-313.35		
Total costs			2119.19	1428.01-2812.16	1813.11	1706.12-1908.35	-306.08	-1011.68 to 385.98	.37

### Organizational Evaluation

**Figure 2:**
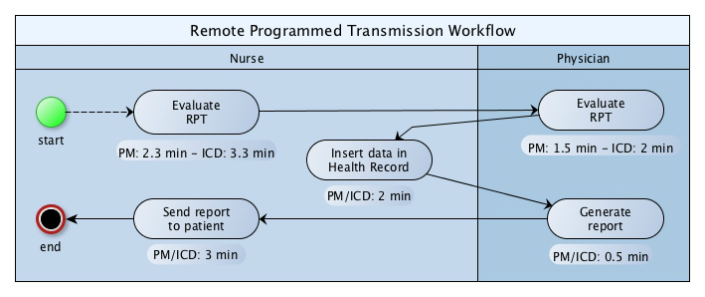
Workflow and workload diagram for implantable cardiac defibrillator (ICD) and pacemaker (PM) remote programmed transmission (RPT).

**Figure 3:**
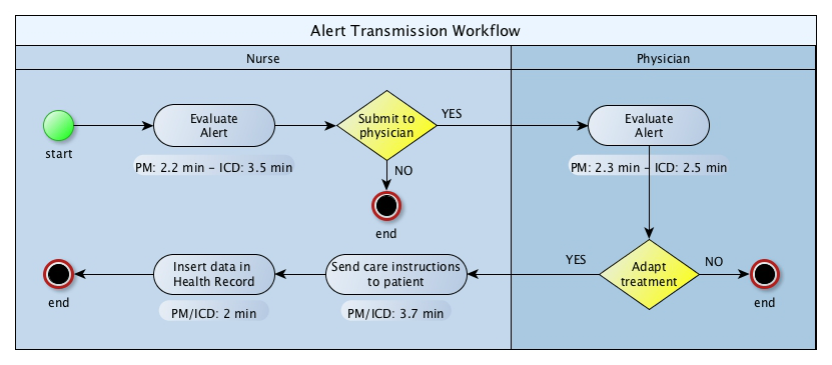
Workflow and workload diagram for implantable cardiac defibrillator (ICD) and pacemaker (PM) alert transmissions.

The organizational analysis focused on the workflow and workload. Figures 2 and 3 show the workflow and workload for the RPT and the alert management activities, respectively.

None of the facilities involved in the study had integrated RM data with the cardiology electronic medical record (EMR), nor had they introduced a software capable to generate a single interface allowing the collection and collation of data from all providers. As a result, staff had to use different portals to access data and manually enter them into the cardiology EMR; this process represented a waste of time and contributed to generate data errors. About 48% (7.3/15.2 minutes per patient per year) of time spent by nurses was used to insert data into the health record and to communicate with the patient. An important reason behind why contacts with patients were related to gateway connecting problems (26.0%, 54/207 for PM and 14.3%, 63/439 for ICD of real events). Nurses filtered 80.0% (768/960) of true or false positive alerts. PMs’ false alert totalled 42.2% (151/358), whereas ICDs’ false alert amounted to 27.1% (163/602).

Table 4 shows the mean time (in minutes) spent by a health care professional (nurse or physician) to provide one-year follow-up to a patient in the I-group versus the U-group. The time to deliver the follow-up in the I-group is reported as the time to offer the telemedicine service only, and as the time spent to carry out RM plus in-clinic follow-up.

**Table 4:** Mean time (minutes) spent by physicians and nurses - usual care versus intervention.

Type of resource	Mean time U-group (min)	Mean time I-group (only telemedicine) (min)	Mean time I-group (telemedicine + in clinic follow-up performed) (min)	Difference (U-group - I-group) (min)
Physician PM	13.1	4.7	9.0	4.1
Nurse PM	18.1	11.2	18.3	-0.2
Physician ICD	32.8	7.5	19.1	13.7
Nurse ICD	44.2	19.6	36.1	8.1

### In-Clinic Follow-Up in U-Group

In the U-group, 74.8% (205/274) of in-clinic follow-ups resulted in no clinical intervention, nor device reprogramming. As expected, reprogramming during routine in-clinic follow-ups occurred less often (14.8%, 31/210) than during the first in-clinic follow-up after the device implantation (30%, 19/64).

## Discussion

### Principal Findings

#### Clinical Observations

The study clarifies some key aspects concerning the management of a RM service delivered to a population of PM/ICD patients. The introduction of CIED RM showed to be highly effective in detecting and managing clinical and technical events with remarkably limited manpower and health resource consumption. Alerts generated by devices were reviewed in a median (SD) time of 1.18 (2.08) days in the PM group, and 1.03 (1.68) days in the ICD group. The number of in-clinic CIED follow-ups was significantly lower in the I-group (-60% for PM and -54% for ICD) without compromising the patients’ clinical status, with a significant reduction in hospitalizations for the PM-group only, and no significant differences in access to the emergency department for either group (PM, ICD). The significant reduction of cardiologic visits (-56% PM and -7% ICD) in the I-group was probably due to the early detection of clinical events, and the prompt adaptation of treatment by phone.

No other study on PM RM, to our knowledge, has evaluated the reduction of hospitalizations for all causes. The Compas Trial [2] observed a significant reduction in hospitalization for atrial arrhythmia and stroke, confirming the efficacy of early detection of atrial fibrillation events. For the ICD-group, the main studies [1,4] that investigated the number of hospitalizations and the frequency of access to the emergency department, or a composite endpoint including both variables, did not show any significant reduction. It would be useful to design a study with a larger ICD population with the aim to evaluate possible significant effects in hospitalization rates for patients with different heart conditions.

The reaction time was very similar to that found by previous analyses. Studies in the literature show a slightly longer time interval, because they measured time from the alert to the clinician’s decision, instead of the time from the alert to the first visualization by clinicians, as in our case. The Home-Guide Registry [7] showed a mean reaction time to alerts of 3 days. The Compas Trial [2] found a substantial improvement in timeliness of response between the I-group and U-group (122 days). For ICD patients, the main results from the literature providing a comparison of reaction time between the I-group and the U-group were, respectively, as follows: 1 versus 35.5 [1]; 4.6 versus 22 [20]; 1.4 versus 24.8 [21]; and 11 versus 183 [22]. Therefore, we conclude that the use of RM of PM/ICD significantly reduces the time from the event to its assessment.

#### Economic Observations

Economic results also showed substantial savings from RM. The reduction of the average cost of treatment per patient in the PM I-group was € 809.76 (*P*<.001) versus € 313.35 (*P*=.55) in the ICD I-group. The cost saving for the ICD group was not significant and less than for the PM group, probably due to the short duration of the follow-up and the limited number of patients in the U-group. We might also suppose that more frequent detection of clinical and technical events in the ICD group, compared to the PM group, have contributed to the above mentioned difference in cost saving. Unfortunately, wide discrepancies between populations’ health and health care systems’ organization, costs, and reimbursements mean that any generalization may result as inaccurate.

#### Organizational Observations

This is the first study, to our knowledge, that has evaluated the overall time spent by health care providers to manage CIED patients (data reviews, patient calls, medical report generations, data entries in health record). Further, this analysis has distinguished between the workload assigned to nurses and physicians. The reduction in time spent by physicians delivering care to PM and ICD patients in the I-group compared with the U-group was apparent. The time required to review a single RPT or alert was similar [23-25], or lower [26,27] to that published by different authors. Nurses, who filtered 80.0% (768/960) of generated alerts, allowed physicians to focus on serious clinical or technical events and other relevant tasks. Confirming other studies [1,2,20,28-30], our analysis showed that the RM service could reduce in-clinic follow-ups that do not require specific interventions by clinicians.

Although all LHAs had been using RM systems for more than 3 years, RM implementation was rather haphazard and fragmented. Recently, this problem was also pointed out by two studies of the European Heart Rhythm Association [12,31] and an Italian nationwide survey [32]. Moreover, cardiology units had not designed a strategy to involve other physicians, especially general practitioners, referring cardiologists, and other specialists. In other words, there was no integrated health care delivery.

Streamlining the process of RM delivery and adjusting the technology would contribute to reduce the waste of time due to manual data entry, false alerts, and gateway connection problems. A centralized eHealth center, that manages RM of patients belonging to different LHAs, could contribute toward the improvement of follow-up, the standardization of patient care, and the optimization of health care resources’ use. This eHealth center could also simplify the division of labor between clinical and administrative/technical staff. A recent study [33] testing a centralized RM model in which one monitoring center screened and filtered daily RM data in PM /ICD patients from nine satellite clinics, concluded that this model is feasible, reliable, safe, and clinically useful.

### Limitations

This is an observational study that did not assign patients to the I-group and the U-group randomly. Furthermore, the U-group was rather small. We used hospitalization rates for all causes instead of atrial fibrillation, stroke, and congestive heart failure. The use of five different vendors devices might have introduced a systematic bias in the assessment of RM performance.

### Conclusions

This study contributes to strengthen current evidence regarding the effectiveness and efficiency of PM and ICD RM in detecting and managing clinical and technical events through limited use of manpower and other health care resources. It also shows that RM is implemented inconsistently, because it is not supported by a solid strategy. This problem is common across national health care systems in Europe. To fully exploit the potential of RM technology, it will be necessary to formulate, implement, and monitor an overall strategy that standardizes the whole process, connects different clinicians, integrates data from different sources into an EMR, and adopts a single platform capable to manage patients monitored by different devices.

## Abbreviations

AF: atrial fibrillation
CIED: cardiac implantable cardiac electronic device
CRF: case report form
DRG: diagnosis related group
EMR: electronic medical record
ICD: implantable cardiac defibrillator
IQR: interquartile range
LHA: local health authority
MAST: model for assessment of telemedicine
PM: pacemaker
RM: remote monitoring
RPT: remote programmed transmission
WRM: wireless remote monitoring
